# Point mutation of V252 in neomycin C epimerase enlarges substrate-binding pocket and improves neomycin B accumulation in *Streptomyces fradiae*

**DOI:** 10.1186/s40643-022-00613-4

**Published:** 2022-12-05

**Authors:** Xiangfei Li, Fei Yu, Fang Wang, Sang Wang, Rumeng Han, Yihan Cheng, Ming Zhao, Junfeng Sun, Zhenglian Xue

**Affiliations:** 1 Engineering Laboratory for Industrial Microbiology Molecular Beeding of Anhui Province, College of Biologic and Food Engineering, Anhui Polytechnic University, 8 Middle Beijing Road, Wuhu, 241000 China; 2 The Key Laboratory of Industrial Biotechnology, Ministry of Education, School of Biotechnology, Jiangnan University, Wuxi, 214122 China

**Keywords:** *S. fradiae*, Neomycin, NeoN, Catalytic mechanism

## Abstract

Neomycin, an aminoglycoside antibiotic with broad-spectrum antibacterial resistance, is widely used in pharmaceutical and agricultural fields. However, separation and purification of neomycin B as an active substance from *Streptomyces fradiae* are complicated. Although NeoN can catalyze conversion of neomycin C to neomycin B, the underlying catalytic mechanism is still unclear. In this study, the genomic information of high-yielding mutant *S. fradiae* SF-2 was elucidated using whole-genome sequencing. Subsequently, the mechanism of NeoN in catalyzing conversion of neomycin C to neomycin B was resolved based on NeoN–SAM–neomycin C ternary complex. Mutant NeoN^V252A^ showed improved NeoN activity, and the recombinant strain SF-2-NeoN^V252A^ accumulated 16,766.6 U/mL neomycin B, with a decrease in neomycin C ratio from 16.1% to 6.28%, when compared with the parental strain SF-2. In summary, this study analyzed the catalytic mechanism of NeoN, providing significant reference for rational design of NeoN to improve neomycin B production and weaken the proportion of neomycin C.

**Graphical Abstract:**

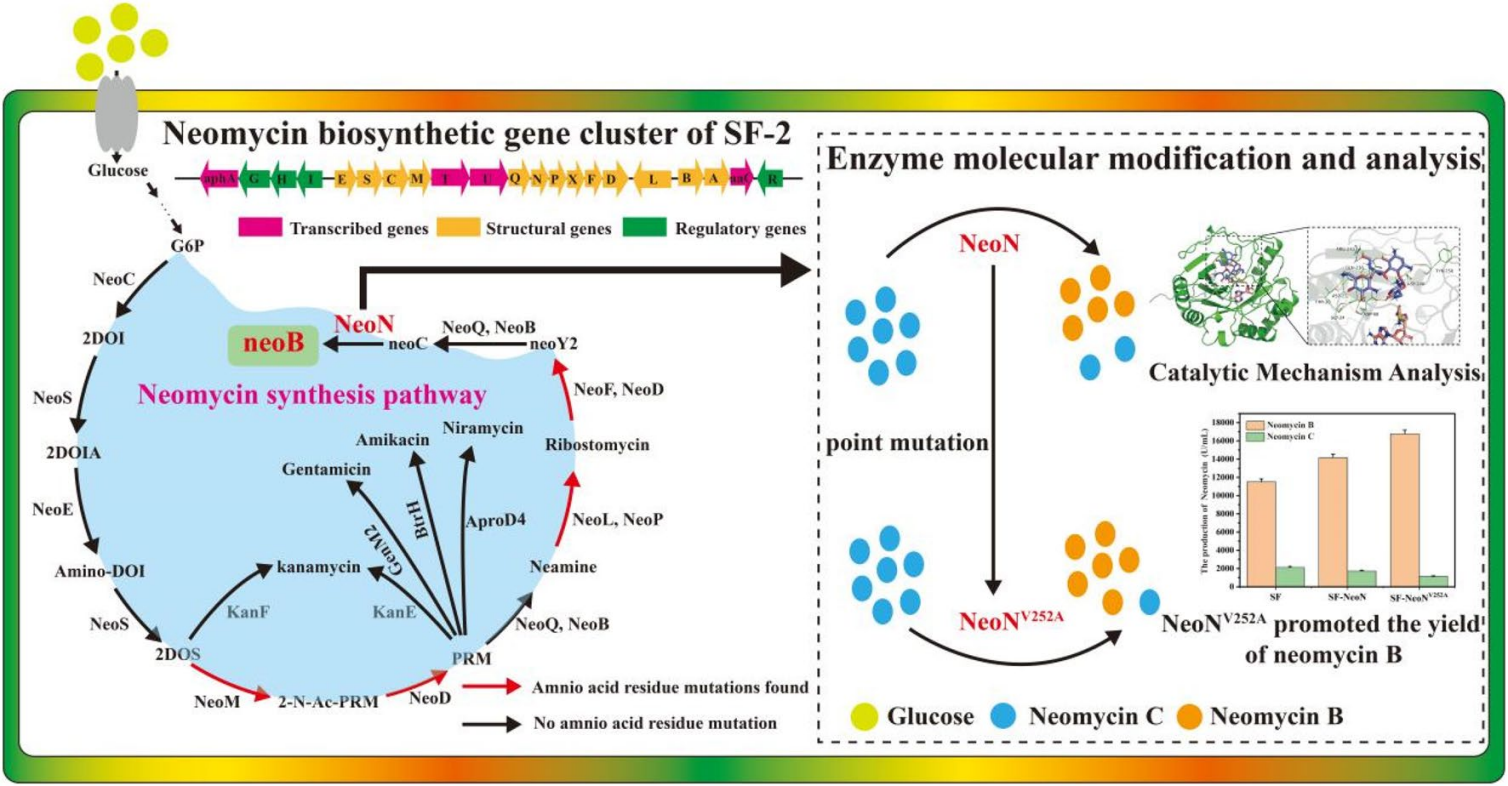

**Supplementary Information:**

The online version contains supplementary material available at 10.1186/s40643-022-00613-4.

## Introduction

Neomycin, the first 2-deoxystreptamine aminoglycoside antibiotic, was isolated from *Streptomyces fradiae* in 1949 (Zheng et al. 2020). Neomycin products were approved by the U.S. Food and Drug Administration (FDA) in 1964, and are widely used in animal husbandry to prevent diseases and improve feed utilization. In recent years, topical products of neomycin, such as neomycin cream, neomycin ophthalmic suspension, and neomycin ointment, have been used to treat human diseases (Hanko and Rohrer 2010). It has been shown that neomycin could inhibit the binding of HIV-related polypeptides to trans-activation response RNA, thereby achieving anti-HIV effects (Chen et al. 2018). In addition, neomycin can activate the inhibitory factor p53 in tumor cells and induce tumor cell apoptosis (Zhang et al. 2019). Besides, neomycin can also be used in combination with other drugs to improve the toxic effect of the drug on lung cancer cells NCI-H460, thereby achieving anticancer effects (Cuccarese et al. 2013; Swiatkowska et al. 2020). Neomycin is a glycoside antibiotic formed by the conversion of carbohydrates through the HMP pathway. It includes three components, A, B, and C, with different chemical structures and biological activities. The antibacterial activity of neomycin mainly involves binding to the 16S rRNA site of the 30S ribosome, which leads to protein mistranslation, inhibition of the assembly of 30S ribosomal subunits, generation of hydroxyl radicals, and suppression of ribonucleases activity, with neomycin B exhibiting the highest antibacterial activity (Homa 2010; Waksman et al. 1949). However, as neomycin B and neomycin C are stereoisomers, the process of separation and purification of neomycin B is relatively complicated. Therefore, metabolic and enzyme engineering strategies must be applied to effectively promote the accumulation of neomycin B and weaken the production of neomycin C as a byproduct.

**Fig. 1:**
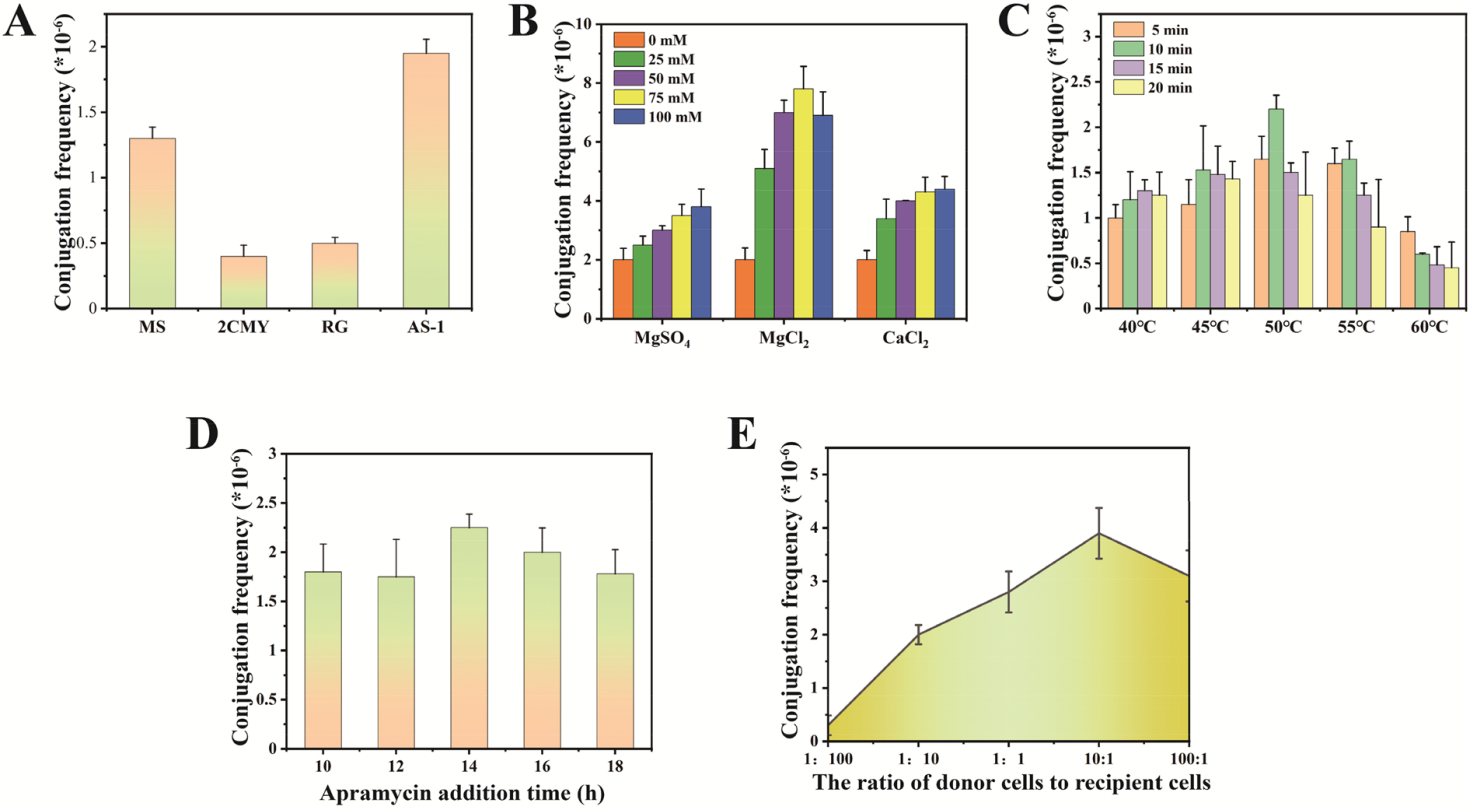
Optimization of the conjugation conditions between strain SF-2 and *E*. *coli*. **A** Effect of **A** medium, **B** metal ions species and concentration, **C** heat shock conditions, **D** antibiotic addition time, and **E** donor:receptor cells ratio on conjugation frequency

Genes involved in neomycin B biosynthesis pathway are mainly distributed in the neo gene cluster. In *S. fradiae*, a total of 12 genes from *neoE* to *neoD* form an operon in the neo gene cluster, including various structural genes involved in the process of neomycin biosynthesis, gene *neoGH*-*aphA* and other regulatory genes form another operon (Zheng et al. 2020) (Fig. 1C). At present, modification of neomycin biosynthesis pathway by molecular biology and other approaches to improve neomycin production has been widely researched. Overexpression of two regulatory genes, *afsA*-g and *neoR,* involved in neomycin B biosynthesis pathway has been reported to increase the production of neomycin to 722.9 ± 20.1 and 564.7 ± 32.5 mg/L, respectively (Meng et al. 2017). Zheng et al. (2019) constructed plasmid PKCZ03 to enhance the expression of neomycin biosynthesis gene cluster and obtained the *S. fradiae* engineering strain SF/PKCZ03 (CGMCC4.576), which exhibited 36% increase in neomycin yield, and addition of key precursors N-acetylglucosamine and l-glutamine resulted in 62–107% increase in neomycin production, respectively. Furthermore, overexpression of the microgene cluster (P_kasO*_-*neoN*-*metK*-P_kasO*_-*neoGH*-*aphA*) in *S. fradiae* CGMCC 4.576 effectively reduced the accumulation of neomycin C from 19.1% to 12.7% and increased the content of neomycin B by about 13.1% (Zheng et al. 2020). At the same time, knockout of NeoN in *S. fradiae* CGMCC 4.576 resulted in the loss of the ability to synthesize neomycin B. Thus, NeoN was elucidated to be the key enzyme in neomycin B synthesis, and overexpression of NeoN could enhance the accumulation of neomycin B and attenuate the production of neomycin C.

NeoN, encoded by *neoN*, is a putative free radical SAM-dependent epimerase that catalyzes the conversion of neomycin C to neomycin B in the final step of neomycin B biosynthesis (Kudo and Eguchi 2016; Kudo et al. 2014). It belongs to the free radical SAM protein family and contains the characteristic CxxxCxxC motif for stabilization of the 4Fe–4S clusters (Besandre et al. 2021; Goldman et al. 2013; Grell et al. 2018). It comprises a unique iron atom that lacks cysteine coordination, and is coordinated by the amino and carboxylic acid moieties of SAM, and this SAM-cluster interaction plays a central role in catalysis (Bauerle et al. 2015; Kuhner et al. 2016). Free radical SAM enzymes have a wide range of catalytic functions, catalyzing steps in metabolism, DNA repair, vitamin and coenzyme biosynthesis, and biosynthesis of many antibiotics such as biotin synthase (BioB), fatty acyl synthase (LipA), pyruvate formatting enzyme (PFL), coporphyrinogen oxidase (HemN), lysine 2,3-amino dismutase (LAM), anaerobic ribonucleotide reductase (ARR), molybdenum pterin biosynthesis enzyme (MoaA), etc. These reactions typically begin with the reductive cleavage of SAM by catalytic iron–sulfur clusters coordinated at the active site of the enzyme, followed by radical-mediated conversion of substrate to product initiated by the resulting l-methionine and 5′-deoxyadenosine radical equivalents. In avilamycin biosynthesis, the free radical SAM enzyme is involved in the related epimerization reaction (Ayikpoe et al. 2019; Grove et al. 2013; Ruszczycky et al. 2018). Similarly, this family of enzymes (2-DOIA dehydrogenase BtrN) is also involved in butirosin biosynthesis pathway, and have been characterized in vitro. By using radioisotope labeling method, Kudo et al. (2014) determined the direction of reaction catalyzed by isomerase NeoN, and found that the enzyme could catalyze the conversion of neomycin C to generate neomycin B. Furthermore, they noted that the reaction occurs at the C-5′ position, and that Cys249 in NeoN provides a hydrogen atom to complete the isomerization of neomycin C during the reaction. Zheng et al. (2020) knocked out *neoN* and confirmed that NeoN catalyzes the conversion of neomycin C to neomycin B, and that overexpression of NeoN could enhance the yield of neomycin B.

In the present study, whole-genome sequencing was first used to analyze the high-yielding neomycin-producing *S. fradiae* strain SF-2, which was obtained by early mutagenesis in the laboratory, and the genetic manipulation system of strain SF-2 was optimized. Then, by overexpressing NeoN and knocking out NeoN in strain SF-2, it was found that NeoN was also a key enzyme for the synthesis of neomycin B. Subsequently, based on the NeoN–SAM–neomycin C ternary complex docking model, the catalytic mechanism of NeoN catalyzing conversion of neomycin C to neomycin B was analyzed. Finally, the recombinant strain SF-2-NeoN^V252A^ was generated, which exhibited 45.8% increase in the yield of neomycin B and a reduction in the proportion of neomycin C to 6.28%, when compared with those noted in the parental strain SF-2. Thus, this study interpreted the whole genome information of high-yielding neomycin-producing mutant strain SF-2, and analyzed the catalytic mechanism of NeoN, providing significant insights for systematic metabolic engineering and rational modification of enzymes to improve neomycin production.

## Results

### Parameters affecting the efficiency of conjugation between *Escherichia coli* and strain SF-2

Conjugation is currently the main genetic manipulation method applied to *S. fradiae*, and the processing conditions have a significant influence on conjugation efficiency. Therefore, in the present study, to improve the conjugation efficiency between *Escherichia coli* and strain SF-2, various parameters, including type and concentration of metal ions, heat shock temperature and time, donor:receptor cells ratio, and antibiotic addition time were exhaustively optimized. As the medium of conjugation must meet the growth requirements of both the donor and receptor cells, four solid media, namely, MS, 2CMY, RG, and AS-1, were selected, and their effects on conjugation were compared. The results showed that the positive transformants on AS-1 medium had the highest frequency of conjugation (Fig. 1A).

Analysis of the effects of MgCl_2_, CaCl_2_, and MgSO_4_ on the conjugation efficiency revealed evident role of all these metal ions in promoting the conjugation efficiency, with MgCl_2_ presenting a higher impact on the frequency of conjugation of strain SF-2. In particular, AS-1 medium supplemented with 75 mM MgCl_2_ exhibited the highest frequency of conjugation (Fig. 1B). As appropriate heat shock treatment of spores could promote spore germination, in the present study, five temperature gradients (40 ℃, 45 ℃, 50 ℃, 55 ℃, and 60 ℃) and four time gradients (5, 10, 15, and 20 min) were selected for improving the frequency of conjugation between *E. coli* and SF-2, respectively. As shown in Fig. 1C, the optimal heat shock temperature was 50 ℃ for 10 min. The ratio of donor and receptor cells can also have an important influence on conjugation efficiency, and the optimal donor:receptor cells ratio can vary for different *S. fradiae* strains. In the present study, a donor:receptor cells ratio of 10:1 produced the highest conjugation frequency. When the number of donor cells was insufficient, the conjugation efficiency was severely affected (Fig. 1D). Similar to the diverse growth rates of different *S. fradiae* strains on conjugation medium, the optimal time of antibiotic addition can also vary. In this study, apramycin was added at 10, 12, 14, 16, and 18 h after conjugation, and the results showed that premature addition of apramycin inhibited the growth of strain SF-2 and was not conducive to conjugation (Fig. 1E). In contrast, late addition of apramycin caused overgrowth of the donor cells and inhibition of the growth of strain SF-2. The frequency of conjugation was the highest when the apramycin addition time was 14 h after conjugation. Finally, based on orthogonal optimization (Additional file 1: Table S2), conjugation frequency was determined to be most affected by donor:receptor cells ratio, followed by MgCl_2_ concentration in the medium, whereas antibiotic addition time had the least effect. Under the optimal combination A_2_B_2_C_2_, the conjugation efficiency reached 14.32 × 10^–6^, which was 7.53-fold higher than that noted under the initial conditions (Additional file 1: Table S3).

### Whole-genome sequencing of high-yielding neomycin-producing mutant strain SF-2

**Fig. 2:**
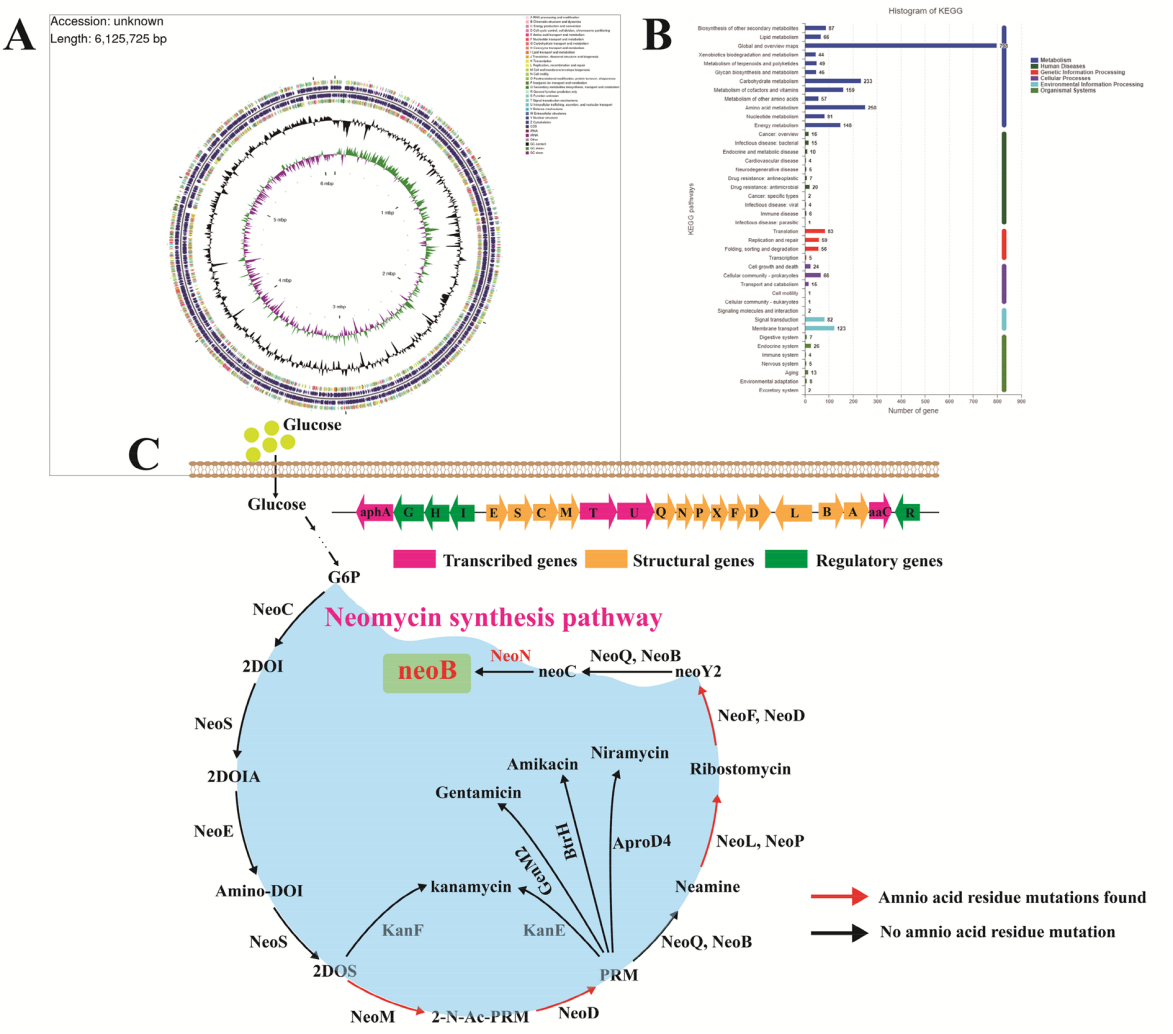
Whole-genome sequencing of strain SF-2. **A** Circos loop map of strain SF-2. **B** Classification of KEGG metabolic pathway of strain SF-2. **C** Neomycin biosynthesis gene cluster of strain SF-2

A high-yielding neomycin-producing mutant strain *S. fradiae* SF-2 was obtained in the laboratory through atmospheric and room temperature plasma mutagenesis (ARTP) via 6 consecutive rounds of mutagenesis screening, and the mutant strain could accumulate 10,849 U/mL neomycin (Yu et al. 2022). In the present study, to further analyze the mechanism of high neomycin production by strain SF-2 and provide accurate theoretical guidance for subsequent systematic metabolic engineering, whole-genome sequencing and assembly of strain SF-2 was performed. As shown in Fig. 2A, the full-length genome of strain SF-2 was 6,127,725 bp, of which the GC content was 74.75%. A total of 6431 protein-coding genes, 62 tRNA genes, and 16 rRNA genes were identified in the genome, and the relevant whole genome sequence information has been uploaded to the NCBI database (GenBank: CP072209.1). The KEGG metabolic pathway classification of SF-2 (Fig. 2B) was mainly divided into six categories: metabolic system, pathogenic system, genetic information processing system, cell differentiation system, environmental information processing, and biological system, with 3309, 97, 239, 114, 214, and 86 annotated genes, respectively. Carbohydrate metabolism and amino acid metabolism accounted for the largest proportions in the metabolic system, indicating that the growth of strain SF-2 required a large amount of carbon and nitrogen sources. The complex metabolic network also revealed the complexity of the strain's metabolic system, revealing that strain SF-2 may accumulate a variety of metabolites. The genetic information processing system mainly included transcription and translation, replication and repair, protein folding, classification, and degradation, which were closely related to the complex morphological differentiation and powerful post-translational modification system of strain SF-2. The strain SF-2 can germinate from spores to form intrabasal hyphae and regenerate into aerial hyphae, and the aerial hyphae finally form mature spores, which involves the assembly and distribution of a large number of DNA and proteins. At the same time, during spore synthesis, the bacteria undergo complex translation. Post-modification systems synthesize complex and diverse metabolites such as virulence factors and antibiotics, which help the organism to compete with other organisms in intricate natural environments. Cell differentiation showed that strain SF-2 not only possessed the cell community characteristics of prokaryotes, such as biofilm formation and a strong quorum sensing system, but also had the eukaryotic local adhesion properties, which facilitated its response to various changes in the natural environment. Furthermore, the environmental information processing system of strain SF-2 revealed that the strain had strong signal transduction and membrane transport functions, especially, the two-component system and ABC transporter. As the basic stimulus–response coupling mechanism, the two-component system can regulate secondary metabolism and morphological differentiation by participating in core processes such as glycolysis, gluconeogenesis, stress signaling pathway, protein secretion, and cell envelope metabolism.

Analysis of genes related to neomycin synthesis gene cluster (Fig. 2C) revealed 30 amino acid residue mutations, one site deletion mutation, and three amino acid insertion mutations (Table 1), which were mainly distributed in NeoM, NeoP, NeoX, NeoF, NeoD, NeoL, and NeoA. These findings suggested that mutation of these genes involved in the neomycin synthesis gene cluster might endow the mutant strain SF-2 with the ability to produce high amount of neomycin, and also provide theoretical guidance for systematic metabolic engineering and rational design to further improve neomycin biosynthesis.

**Table 1:** Strains and plasmids used in this study

	Characteristics	Source
Strains		
*E*. *coli* DH5α *E*. *coli* BL21	General cloning host General expressing host	The lab The lab
*E*. *coli* ET12567	Demethylated strain containing pUZ8002 plasmid for conjugative transfer with actinomycetes	The lab
SF-2	*Streptomyces fradiae*, neomycin-producing strains induced by ARTP	(Yu et al. 2022)
BL21/pET28a	*E*. *coli* BL21 derivative harboring pET28a	The lab
BL21/pET28a-NeoN	*E*. *coli* BL21 derivative harboring pET28a-NeoN	The lab
BL21/pET28a-NeoN^E34A^	*E*. *coli* BL21 derivative harboring pET28a-NeoN^E34A^	This study
BL21/pET28a-NeoN^T38A^	*E*. *coli* BL21 derivative harboring pET28a-NeoN^T38A^	This study
BL21/pET28a-NeoN^D68A^	*E*. *coli* BL21 derivative harboring pET28a-NeoN^D68A^	This study
BL21/pET28a-NeoN^K36A^	*E*. *coli* BL21 derivative harboring pET28a-NeoN^K36A^	This study
BL21/pET28a-NeoN^S251A^	*E*. *coli* BL21 derivative harboring pET28a-NeoN^S251A^	This study
BL21/pET28a-NeoN^V252A^	*E*. *coli* BL21 derivative harboring pET28a-NeoN^V252A^	This study
ET12567/PPR-NeoN	*E*. *coli* ET12567 derivative harboring PPR-NeoN	This study
ET12567/PPR-NeoN^V252A^	*E*. *coli* ET12567 derivative harboring PPR-NeoN^V252A^	This study
ET12567/PKC1139-NeoN	*E*. *coli* ET12567 derivative harboring PKC1139-NeoN	This study
SF-2-NeoN	SF-2 derivative the expression of NeoN	This study
SF-2-NeoN^V252A^	SF-2 derivative the expression of NeoN^V252A^	This study
SF-2ΔNeoN	SF-2 derivative the deletion of *neoN*	This study
*Plasmids*		
pET28a	Kan^r^; Expression vector for the expression of target protein	The lab
PPR (pSET152-PermE*)	*E. coli*–*S. fradiae* shuttle vector for the expression of target protein	The lab
pKC1139	*E. coli*–*S. fradiae* shuttle vector for the deletion of target protein	The lab
pET28a-NeoN	Derived from pET28a, for induced expression of NeoN	This study
pET28a-NeoN^E34A^	Derived from pET28a, for induced expression of NeoN^E34A^	
pET28a-NeoN^T38A^	Derived from pET28a, for induced expression of NeoN^T38A^	This study
pET28a-NeoN^D68A^	Derived from pET28a, for induced expression of NeoN^D68A^	This study
pET28a-NeoN^K36A^	Derived from pET28a, for induced expression of NeoN^K36A^	This study
pET28a-NeoN^S251A^	Derived from pET28a, for induced expression of NeoN^S251A^	This study
pET28a-NeoN^V252A^	Derived from pET28a, for induced expression of NeoN^V252A^	This study
PPR-NeoN	Derived from PPR, for the expression of NeoN	This study
PPR-NeoN^V252A^	Derived from PPR, for the expression of NeoN^V252A^	This study
PKC1139-ΔNeoN	Derived from PKC1139, for the deletion of *neoN*	This study

### Effects of NeoN on neomycin biosynthesis

**Fig. 3:**
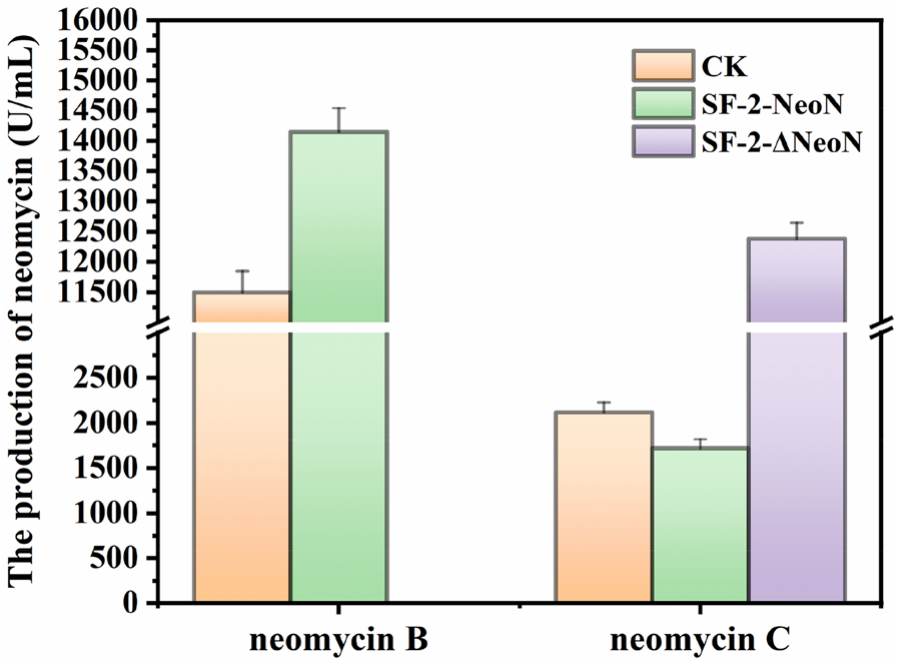
Production of neomycin B and neomycin C by strains SF-2, SF-2-NeoN, and SF-2ΔNeoN during fermentation

Neomycin includes three components A, B, and C with different chemical structures and biological activities (Zheng et al. 2020). Among them, component A is extremely small, component B has the highest antibacterial activity, and component C is more toxic (about 300-fold) than component B and is considered to be the main impurity. As neomycin B and neomycin C are stereoisomers, it is difficult to separate them in industry. NeoN, encoded by *neoN,* is a SAM-dependent epimerase that catalyzes the conversion of neomycin C to neomycin B, thereby enriching the production of neomycin B and attenuating the accumulation of neomycin C; thus, enhancing the expression of NeoN can increase the proportion of neomycin B in the final product (Kudo et al. 2014). In the present study, to verify whether NeoN also affects the proportion of neomycin B in strain SF-2, NeoN overexpression strain SF-NeoN and NeoN knockout strain SF-2ΔNeoN were constructed based on the optimized conjugation system, respectively, and the accumulation of neomycin B was determined by fermentation. The results showed that strain SF-NeoN could accumulate 14,149 U/mL neomycin B, which was 23.07% higher than that produced by the parental strain SF-2, whereas strain SF-2ΔNeoN essentially lost the ability to synthesize neomycin B, thus indicating that NeoN is crucial for the synthesis of neomycin B in strain SF-2, consistent with the findings of previous studies (Fig. 3).

### Analysis of the catalytic mechanism of NeoN

**Fig. 4:**
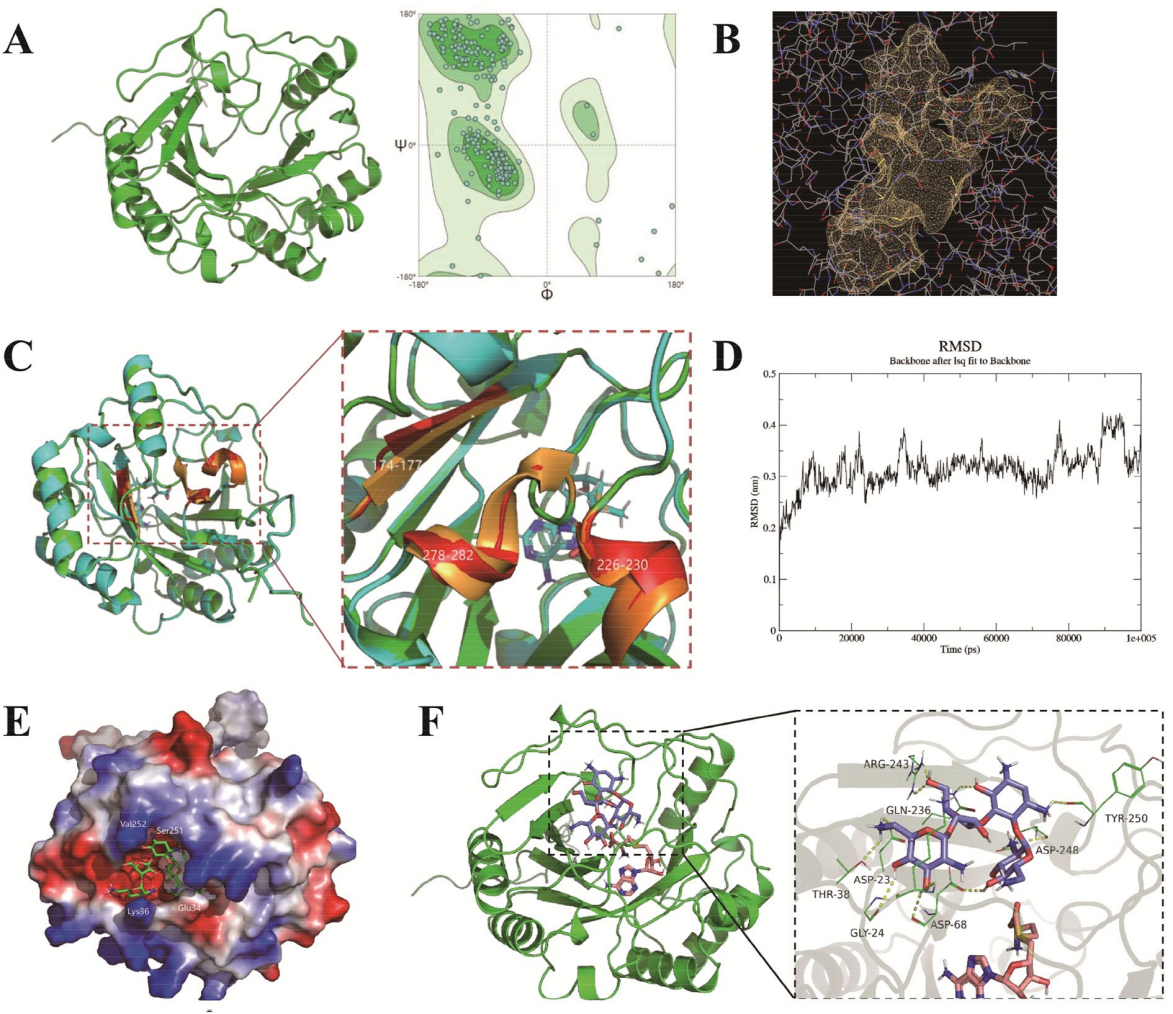
Catalytic mechanism of NeoN. **A** Tertiary structure of NeoN. **B** Catalytic pockets of NeoN computed by DoGSiteScorer. **C** Comparison of the structure of NeoN with that of NeoN–SAM complex. **D** RMSD of the NeoN–SAM backbone. **E** Surface electrostatic potential energy of NeoN–SAM–neomycin C complex. **F** Docking of NeoN–SAM with neomycin C

To further analyze the catalytic mechanism of NeoN in the conversion of neomycin C to neomycin B, three-dimensional structural model of NeoN was established by using AlphaFold prediction (Fig. 4A). As shown in the Ramachandran diagram (Fig. 4A), 91.82% of the amino acid residues clustered in the optimal area (green area), implying that this protein model was reasonable and could be used for subsequent research. Subsequently, the CDD database of NCBI was used to predict the structure domain of the protein model, and its *E*-value was observed to be less than the threshold value of 10^–6^, demonstrating that the prediction of structure domain was reliable. The amino acids in positions 20–189 of the sequence belonged to the free radical dependent SAM superfamily, and those in positions 26–33 were the binding sites for the formation of iron–sulfur clusters and SAM. The cysteine site of this conserved sequence CxxxCxxC coordinates with the 4Fe–4S cluster to jointly transfer an electron from the iron–sulfur cluster to SAM, resulting in the reductive cleavage of SAM to methionine and 5'-deoxyadenosine, which extract a hydrogen atom from the appropriate position, and the SAM is either consumed or recovered and reused in the process.

The “catalytic pocket” with a volume of 912.64 Å^3^ formed by Ile28–Leu181 in the space of NeoN was computed by using the online software DoGSiteScorer (Fig. 4B and Table 2), and the results revealed that this catalytic pocket may be utilized for binding with SAM and substrate molecules. Therefore, the small molecule ligand SAM and receptor NeoN were docked through Discovery Studio (DS) to obtain the NeoN–SAM complex (Fig. 4C). The docked SAM molecules were noted to bind around the sites of Cys26, Cys30, and Cys33 located in the conserved sequence CxxxCxxC, which are used to form 4Fe–4S clusters, with a binding energy of − 7.1 kcal/mol. The obtained NeoN structure was overlapped with the composite NeoN–SAM structure (Fig. 2D). The RMSD value of the NeoN structure before and after docking with SAM was about 0.216 Å, indicating that the overall structure of NeoN and NeoN–SAM was similar. With the docking of SAM to NeoN, three distinct regions flanking the binding region, namely, region 1 (L174–F177), region 2 (E278–S282), and region 3 (D226–N230), were detected. In the NeoN–SAM complex, the β-sheet of region 1 was folded and expanded outward, while the loop region of regions 2 and 3 was significantly increased; all these changes increased the internal space of the catalytic pocket. Combined with the analysis of surface electrostatic potential energy, it was found that the original acidic environment in the catalytic pocket became a neutral environment after docking of SAM (Fig. 4E). In summary, the change in NeoN after binding to SAM increased the internal space of NeoN and altered the catalytic environment, which was more conducive to the recognition and binding of the substrate neomycin C and provided conditions for the subsequent entry and binding of neomycin C molecules.

**Table 2:** Whole-genome sequencing of strain SF-2 with respect to neomycin biosynthesis pathway

Genes	Mutation base	Mutation amnio acid residue
NeoE	T91C, C112T, G895A	None
NeoS	G373A, C652T	None
NeoC	T412C, T958G, G1018T	None
NeoM	G247C, C585T, A1115G	V49A, A226T
NeoQ	C43G, C1366G	None
NeoN	G667A	None
NeoP	G241T, C261T, C448T, G518C	A62G, A148T
NeoX	G46C, C176T	C26Y
NeoF	T14C, C69T, A265C, G795T, C931T	A345T, E363G
NeoD	G108C, A205C,G332C, C470T, T501A, C832T	T113S, P170R, G124D, P245A
NeoL	G142C, T347C, T362C, G395C, C403G, G428A, C510G, T834G, C930T, A1485G, T1599C, A1602G, G1608C, T1659A, C1918G	E48Q, V116A, V121A, Q135E, R143H, P640A
NeoB	G61C, C475G, G1092C, G1219A	None
NeoA	T161C, G283C, A376T, G461A, G859C, T991C, C1111T, G1341C, G2099C, T2151C, G3396A, 2088 Del, 2113 Del, 2125 Del, 3649_3650 Indel CCCGGTGTT	P163S, T578A, R589V, G590A, S591V, P592A, A593R, W594L, T595E, R596A, P597A, R848G, A1141V, D1241G, 70_71 Indel GTP, 588 Del
NeoR	T876C, T995A, C1089G, G1272C, T1529C, C1617T, A1671G, T1989C, C2319G, C2331T, T2373C, G2374C, G2644A	V332D, T510I, G792R, E882K, 866_867 Indel LAAA
NeoG	G597C, C647T, G696A	A216V
NeoH	C81G	None
NeoI	None	None

Furthermore, DS was used to dock neomycin C to obtain the NeoN–SAM–neomycin C ternary complex (Fig. 4F). Analysis of the structure of NeoN–SAM–neomycin C ternary complex by surface potential distribution map revealed that the presence of several amino acid residues (Glu34, Lys36, Ser251, Val252, etc.) in the binding pocket made the surface of the protein to bulge, thus narrowing the entrance. The SAM molecule penetrated deep into the binding pocket where the SAM molecule binding cavity existed, while the neomycin C molecule was tightly bound in the NeoN binding pocket near the entrance. Binding force analysis showed that neomycin C was adjacent to the SAM molecule and tightly bound to the catalytic pocket. A total of 14 amino acid residues were detected within 3 Å of the neomycin C molecule in the NeoN substrate-binding pocket, and the amino acid residues capable of achieving hydrogen bond interactions included Thr38, Gly24, Asp68, Asp23, Gln236, Arg243, Asp248, and Tyr250 (Fig. 4F). These hydrogen bonds were the key to the binding of neomycin C molecule to the NeoN–SAM complex, which stabilized the conformation of neomycin C. Unlike other amino acid residues, Asp68 could form three hydrogen bonds and two salt bridges with the neomycin C molecule and Thr38 could form three hydrogen bonds with the neomycin C molecule, contributing to the binding of NeoN to the substrate. Furthermore, the presence of Val252 could make the protein surface at the substrate-binding pocket to bulge, thus narrowing the entrance for substrate binding (Fig. 4E), which produced an unfavorable molecular binding force. These results provide significant insights for rational transformation of NeoN to promote neomycin B accumulation.

### NeoN^V252A^ promoted the accumulation of neomycin B

**Fig. 5:**
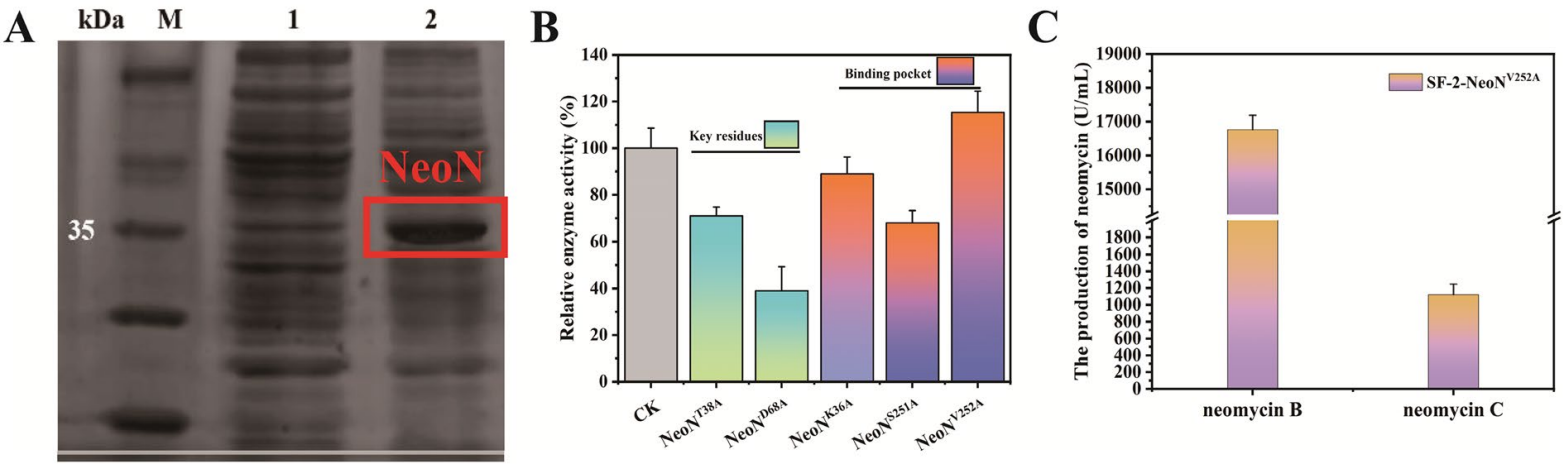
Effects of NeoN mutant on neomycin production. **A** Expression of NeoN in *E*. *coli* BL21. **B** Relative enzyme activities in NeoN mutants. **C** Production of neomycin B and neomycin C by strain SF-2-NeoN^V252A^ during fermentation

The NeoN–SAM–neomycin C ternary complex model revealed Asp68 and Thr38 as the major contributors to the binding of NeoN to the substrate. Therefore, to investigate their role in the catalytic reaction of NeoN, these two amino acids were mutated to the uncharged hydrophobic amino acid, alanine, which had less influence on the protein structure. It must be noted that neomycin C is a large molecule composed of four sugar rings, whereas the catalytic pocket of NeoN is both small and narrow, with protruding amino acid side chain at the entrance making it difficult for neomycin C to enter. Hence, in the present study, Glu34, Lys36, Ser251, and Val252 at the entrance of the binding pockets were mutated into alanine with methyl side chain and low steric hindrance to increase the area and volume of the substrate-binding pockets and allow the entry of more substrate molecules into the pockets for catalytic reactions. Heteroexpression of NeoN and its mutants in *E. coli* BL21 (Fig. 5A) and subsequent analysis of the enzyme activities in the wild-type NeoN and its mutants showed decreased enzyme activities in the mutants NeoN^T38A^ and NeoN^D68A^, when compared with that in the wild-type NeoN. In particular, the relative enzyme activity in NeoN^T38A^ and NeoN^D68A^ was 72–39% lower than that noted in the wild-type NeoN, respectively. With regard to mutants with altered catalytic pocket, except for NeoN^E34A^, which showed no enzyme activity, NeoN^K36A^ and NeoN^S251A^ exhibited varying degrees of decrease in the relative enzyme activities (87.5–66.9%, respectively). In contrast, the relative enzyme activity in mutant NeoN^V252A^ was 115.2% higher than that observed in the wild-type NeoN (Fig. 5B). Analysis of the catalytic pocket with DoGSiteScorer revealed an increase in the volume of the catalytic pocket of NeoN^V252A^ by 51.01 Å^3^, when compared with that of NeoN (Table 2). Therefore, it was speculated that NeoN^V252A^ could expand the catalytic pocket, which facilitated the entry and binding of the substrate molecule neomycin C. Subsequently, recombinant strain SF-2-NeoN^V252A^ was constructed by overexpressing NeoN^V252A^ in strain SF-2. The engineered strain SF-2-NeoN^V252A^ was able to accumulate 16,766.6 U/mL neomycin B, which was 18.5% higher than that produced by SF-2-NeoN (Fig. 5C).

## Discussion

The high-yielding neomycin-producing strain SF-2, obtained through ARTP via 6 consecutive rounds of mutagenesis screening, has been reported to accumulate 10,849 U/mL neomycin (Yu et al. 2022). In the present study, strain SF-2 was analyzed by whole-genome sequencing and its genetic manipulation system was optimized. Subsequently, the catalytic mechanism of NeoN catalyzing the conversion of neomycin C to neomycin B was investigated, and the constructed recombinant strain SF-2-NeoN^V252A^ produced 45.8% higher neomycin B, when compared with that generated by the strain SF-2, and the proportion of neomycin C decreased to 6.28% relative to that produced by the parental strain.

With rapid technological development, the cost of whole-genome sequencing has significantly reduced. At present, a large number of microbial genome data have been widely published and reported, which provide vast information for the functional analysis of microbial genes (Choo et al. 2016; Sandoval et al. 2015), and these studies make significant contributions to public health (Kwong et al. 2015), epidemiology (Llarena et al. 2017), health economics (Frank et al. 2013), etc. Genetic engineering and other methods, which modify related genes and metabolic pathways in cells to improve the yield and synthesis efficiency of specific metabolites, have currently become a very important approach for producing industrial strains of *Streptomyces* (Xu et al. 2022). Sequencing, annotation, and analysis of the *S. fradiae* genome could help to understand its genetic variation principles and important metabolic pathways and regulatory mechanisms at the molecular level, and provide a theoretical basis for the construction of high-yielding strains. In addition, discovery of biosynthetic gene clusters of important metabolites in the *Streptomyces* genome is crucial for antibiotic development, synthetic biology research, etc. In the present study, the whole genome of strain SF-2 was sequenced and assembled, and a complete genome sequence with a length of 6,127,725 bp was obtained, which can provide a theoretical basis for the construction of neomycin engineering strains. Furthermore, point mutations of NeoM, NeoP, NeoX, NeoF, NeoD, NeoL, and NeoA in the neomycin synthesis pathway gene cluster, and neomycin synthesis regulation gene NeoR were identified, suggesting that mutations in these key enzymes may play an important role in neomycin synthesis. It has been reported that the transcription factor NeoR controls the biosynthesis of neomycin by regulating NeoG and NeoH. Hence, in future research, the catalytic mechanism of key enzymes in neomycin synthesis gene cluster needs to be explored to further improve the level of neomycin synthesis in strain SF-2. As transcription factors play an important role in cell metabolism by regulating the metabolic network (Li et al. 2021), NeoR could be overexpressed in strain SF-2 and comparative transcriptomic analysis of the key genes regulated by NeoR and the interaction between NeoR and its binding promoters by EMSA experiments could help to regulate neomycin biosynthesis.

In the present study, the catalytic mechanism of NeoN was resolved and the developed recombinant strain SF-2-NeoN^V252A^ accumulated 16,766.6 U/mL neomycin B, which was 45.8% higher than that produced by strain SF-2, and the proportion of neomycin C decreased from 16.1% to 6.28% relative to that generated by the parental strain. It has been reported that the higher enzymatic activity of NeoN could promote neomycin B biosynthesis and reduce the proportion of neomycin C in *S. fradiae* (Zheng et al. 2020). However, in the present study, the enzyme activity in the mutant NeoN^V252A^ was only increased by 115.2%, when compared with that in NeoN. Therefore, to further increase neomycin B biosynthesis, the enzyme activity in NeoN could be enhanced by site-directed saturation mutation of V252. Furthermore, it was noted that the volume and surface area of the catalytic pocket of mutant NeoN^S251A^ significantly increased, whereas the enzyme activity significantly decreased. This may be owing to the obvious enhancement of the surface area of the catalytic pocket, which reduced its binding specificity, thereby decreasing the enzyme activity. Hence, the surface area and volume of the binding pocket could possibly be increased by mutating S251 with higher steric hindrance, which may be more conducive to maintain the catalytic specificity of NeoN and promote the enzymatic activity of NeoN, thereby enhancing neomycin biosynthesis.

## Conclusions

This study elucidated the whole genome information of the high-yielding neomycin-producing *S. fradiae* strain SF-2, and determined the catalytic mechanism of NeoN. The constructed recombinant strain SF-2-NeoN^V252A^ accumulated 16,766.6 U/mL neomycin B, which was 45.8% higher than that produced by strain SF-2, and the proportion of neomycin C decreased from 16.1% to 6.28% relative to the parental strain. Besides, clarification of the catalytic mechanism of NeoN provides crucial insights for rational designing of NeoN to improve neomycin B production and reduce the proportion of neomycin C.

## Methods

### Strains, plasmids, and growth conditions

The strains and plasmids used in this study are listed in Table 3, and the primers employed in this study are presented in Supplementary Materials (Additional file 1: Table S1). *S. fradiae* SF-2, *E. coli* DH5α, *E. coli* BL21, and *E*. *coli* ET12567 were deposited in the laboratory. *E*. *coli* DH5α and *E*. *coli* BL21 cells were used as hosts for cloning and expression of target protein, respectively. *E*. *coli* ET12567 was employed for conjugative transfer with Actinomycetes. *S. fradiae* SF-2 was cultivated on AS-1 solid medium or in YEME liquid medium at 35 ℃. *E. coli* DH5α, *E. coli* BL21, and *E*. *coli* ET12567 cells were grown in LB medium at 37 ℃. *S. fradiae* SF-2 was first cultivated in seed medium at 35 ℃ and 260 rpm to log phase (40–50 h), and then inoculated (1% *v*/*v*) into the fermentation medium at 35 ℃, 260 rpm, and 75% relative humidity for 7 days.

**Table 3:** Information on the catalytic pocket of each mutant

Size and shape descriptors	Volume[Å^3^]	Surface[Å^2^]	Depth[Å]
NeoN	912.64	1370.45	22.03
NeoN^E34A^	694.91	1053.27	19.94
NeoN^K36A^	818.18	1498.79	21.50
NeoN^S251A^	1349.63	1850.20	30.82
NeoN^V252A^	963.65	1220.57	19.50

The composition of MS medium can be found in a previous study (Huang et al. 2002). The AS-1 medium contained the following (g/L): yeast powder, 1; l-alanine, 0.2; l-arginine, 0.2; l-aspartate, 0.5; NaCl, 2.5; Na_2_SO_4,_ 10; soluble starch, 5; agar power, 20, and pH, 7.3–7.8. The seed medium was composed of the following (g/L): (NH_4_)_2_SO_4,_ 1; yeast powder, 20; groundnut meal, 10; soluble starch, 10; glucose, 30; corn steep liquor, 10; trypsin, 5; Na_2_HPO_4,_ 1; CaCO_3,_ 10; bean oil, 2; and pH, 7.3–7.8. Fermentation medium contained the following (g/L): soluble starch, 70; groundnut meal, 28; yeast powder, 6; (NH_4_)_2_SO_4,_ 6; glucose, 20; corn steep liquor, 2.5; trypsin, 9; medium-temperature bean cake powder, 5; NaCl, 4.5; high-temperature amylase, 0.3; Na_2_HPO_4_, 0.4; CaCO_3_, 4; bean oil, 3; and pH, 6.8–7.3.

### Whole-genome sequencing

The strain SF-2 (preserved at – 80 ℃) was cultured on MS medium at 30 ℃ for 5–7 days. Then, single colonies were picked and inoculated into seed medium and cultivated at 35 ℃ and 260 rpm to logarithmic phase. Subsequently, the cultured cells were collected, centrifuged at 8000 rpm and 4 ℃ for 10 min, quickly frozen with liquid nitrogen, and sent to Shanghai Megji Biomedical Technology Co in dry ice for whole-genome sequencing. For annotation, *S. fradiae* DSM 40,063 (GenBank: AJ629247.1) was used as the reference. Glimmer (Delcher et al. 2007), GeneMarkS (Besemer and Borodovsky 2005), and Prodigal software were used to predict the coding sequence in the genome, and tRNAscan-SE (Chan and Lowe 2019) software was employed to obtain nucleotide sequence information, anticodon information, and secondary structure information of the tRNA in the genome of each sample. Barrnap software was used to acquire species, location, and sequence information of all the rRNA in the genome of each sample.

### Construction of plasmids and recombinant strains

To construct recombinant plasmid PPR-NeoN, the target fragment NeoN was amplified with the genome of strain SF-2 as template and primer pair NeoN F/NeoN R. T4 DNA ligase (TaKaRa) was employed to connect the obtained target DNA fragment and the linearized vector pSET152 (PPR), both of which were digested and purified with *Not*I and *Eco*RV. To generate recombinant plasmid pET28a-NeoN, the target fragment was amplified using the genome of strain SF-2 as template and primer pair 28a-NeoN F/28a-NeoN R. Then, T4 DNA ligase (TaKaRa) was utilized to connect the obtained target DNA fragment and the purified linearized vector pET28a, both of which were digested with *Eco*RI and *Xho*I. To construct recombinant plasmid pET28a-NeoN^V252A^, linearized fragment was amplified using the recombinant plasmid pET28a-NeoN as template and primer pair V252A-F/V252A-R. *Dpn*I was used to digest the template and ClonExpress II One Step Cloning Kit (Vazyme, Nanjing, China) was employed to connect the obtained target DNA fragment.

The other five NeoN mutants were constructed in the same way as pET28a-NeoN^V252A^, with corresponding primer pairs. To construct recombinant plasmid PPR-NeoN^V252A^, the target fragment NeoN^V252A^ was amplified with the plasmid pET28a-NeoN^V252A^ as template and primer pair NeoN F/NeoN R. Then, T4 DNA ligase (TaKaRa) was used to connect the obtained target DNA fragment and the linearized vector pSET152 (PPR), both of which were digested and purified with *Not*I and *Eco*RV. To generate recombinant plasmid PKC1139-ΔNeoN, the target fragment ΔNeoN was amplified with the genome of SF-2 as template and primer pair ΔNeoN F/ΔNeoN R. T4 DNA ligase (TaKaRa) was used to connect the obtained target DNA fragment and the linearized vector pKC1139, both of which were digested and purified with *Xba*I and *Bam*HI. All the constructed recombinant plasmids were transformed into *E*. *coli* DH5α cells and identified by colony PCR and sequencing.

### Conjugation and optimization between *E*. *coli* and SF-2

Preparation of donor cells: The donor *E*. *coli* ET12567 cells (preserved at − 80 ℃) were cultured on LB medium at 37 ℃, and single colonies were picked and inoculated into LB medium at 37 ℃ and 180 rpm to an optical density (OD_600_) of 0.4–0.6. Then, the cells were collected by centrifugation at 8000 rpm for 10 min at 4 ℃, re-suspended, and washed twice with sterile saline to maintain the cell concentration at about 10^8^ cfu/mL.

Preparation of receptor cells: Strain SF-2 (preserved at − 80 ℃) was cultured on MS medium at 30 ℃ for 5–7 days. The mature spores were washed with an appropriate amount of YEME liquid medium and dispersed using sterile glass beads with sufficient oscillation. Subsequently, the hyphae were removed by filtration using 8 layers of gauze to obtain a spore suspension, with spores concentration adjusted to 10^8^ cfu/mL.

Conjugation method: The spore suspension of strain SF-2 was heated at 50 ℃ for 10 min and preincubated at 37 ℃ and 200 rpm for 3 h. Then, the donor cells were mixed with cultured spore suspension of SF-2 (donor:receptor cells = 10:1), centrifuged at 4000 rpm for 5 min at 4 ℃, and spread on AS-1 plate with 75 mM MgCl_2_ at 30 ℃ for 14 h. Next, 50 μg/mL apramycin and 500 μg/mL nalidixic acid were poured onto the AS-1 plate and incubated at 30 ℃ for another 4–5 days. The frequency of conjugation was calculated as the number of zygotes/number of initial spores.

### Bioinformatics tools

AlphaFold database (https://alphafold.com/) was used to obtain the 3D structural model of NeoN. The VERIFY_3D Server (http://services.mbi.ucla.edu/Verify_3D/) was employed to check the quality of the generated model. CDD database (https://www.ncbi.nlm.nih.gov/Structure/cdd/cdd.shtml) and Uniport (https://www.uniprot.org/) were utilized to analyze the classes, domains, and characteristic amino acid positions of the protein superfamily to which NeoN belongs. DoGSiteScorer was used to compute the protein catalytic pocket, DS was employed to examine the interactions, and Gromacs 2019.5 was utilized for molecular dynamics simulations. The simulation temperature was 300 K and Gromacs’ all-atom force field and SPC water model were selected.

### Analytical methods

Spectrophotometer (UV-1800) was used to determine OD_600_. Detection of neomycin B and neomycin C was performed as described earlier (Zheng et al. 2020), and the NeoN enzyme activity was assayed as mentioned previously (Kudo et al. 2014). All the assays were performed in triplicate.

### Supplementary Information

**Additional file 1: Table S1.** Primers in this study. **Table S2.** Orthogonal factor level table. **Table S3.** Results and data analysis of orthogonal test.

## Data Availability

The datasets used and/or analyzed in this study are available from the corresponding author on reasonable request.
