# Supplementary material for: Point mutation of V252 in neomycin C epimerase enlarges substrate-binding pocket and improves neomycin B accumulation in Streptomyces fradiae
Source: Bioresour Bioprocess. 2022 Dec 5;9(1):123. doi: 10.1186/s40643-022-00613-4 (PMC10991966; doi:10.1186/s40643-022-00613-4)
Supplement: Supplementary file 1 — Additional file 1: Table S1. Primers in this study. Table S2. Orthogonal factor level table. Table S3. Results and data analysis of orthogonal test. [file 40643_2022_613_MOESM1_ESM.docx]

**Table S1** Primers in this study

| Primers name | sequences | Illustrate |
| --- | --- | --- |
| NeoN F | ATAGCGGCCGCGATGACCACCGAC | *NotI* |
| NeoN R | CGAGATATCTCATACGAGCG | *EcoRV* |
| ΔNeoN F | GCTCTAGAAGAAGACCAAGGTGGACCAG | *XbaI* |
| ΔNeoN R | CGCGGATCCGAGTAGCAGTCGC | *BamHI* |
| 28a-NeoN F | CGGAATTCATGACCACCGACATCGT | *EcoRI* |
| 28a-NeoN R | CCCCTCGAGTCATACGAGCGGCAG | *XhoI* |
| T38A-F | ggtcaagaagGCCaaggtggaccagccc |  |
| T38A-R | ggtccaccttGGCcttcttgacctcgcagtag |  |
| D68A-F | ggtggagtccGCCggcgagatcaccctct |  |
| D684-R | ggcgagatcaGGCggactccacccggaac |  |
| E34A-F | cacctactgcGCGgtcaagaagaccaaggtggac |  |
| E34A-R | tcttcttgacCGCgcagtaggtgcaccgga |  |
| K36A-F | ctgcgaggtcGCGaagaccaaggtggaccag |  |
| K36A-R | ccttggtcttCGCgacctcgcagtaggtg |  |
| S251A-F | cgactgctacGCGgtgcccaagcacctg |  |
| S252A-R | gcttgggcacCGCgtagcagtcgcacttgac |  |
| V252A-F | actgctactcGCGcccaagcacctggtg |  |
| V252A-R | ggtgcttgggCGCcgagtagcagtcgcac |  |

**Table S2** Orthogonal factor level table.

| Level | A: MgCl_2_ (mM) | B: Donor cell : recipient cell | C: Apramycin addition time |
| --- | --- | --- | --- |
| 1 | 65 | 5:1 | 13 |
| 2 | 75 | 10:1 | 14 |
| 3 | 85 | 15:1 | 15 |

**Table S3** Results and data analysis of orthogonal test.

| Test No. | A | B | C | Conjugation frequency (×10^-6^) |
| --- | --- | --- | --- | --- |
| 1 | 1 | 1 | 2 | 7.25 |
| 2 | 1 | 2 | 3 | 10.59 |
| 3 | 1 | 3 | 1 | 8.43 |
| 4 | 2 | 1 | 3 | 11.26 |
| 5 | 2 | 2 | 2 | 14.32 |
| 6 | 2 | 3 | 1 | 8.78 |
| 7 | 3 | 1 | 2 | 9.66 |
| 8 | 3 | 2 | 1 | 10.46 |
| 9 | 3 | 3 | 3 | 9.98 |
| k1 | 8.757 | 9.390 | 9.223 |  |
| K2 | 11.453 | 11.790 | 10.117 |  |
| K3 | 10.033 | 9.063 | 10.610 |  |
| R | 2.697 | 2.727 | 1.387 |  |
